# Use and Outcomes of Dual Chamber or Cardiac Resynchronization Therapy Defibrillators Among Older Patients Requiring Ventricular Pacing in the National Cardiovascular Data Registry Implantable Cardioverter Defibrillator Registry

**DOI:** 10.1001/jamanetworkopen.2020.35470

**Published:** 2021-01-26

**Authors:** Ryan T. Borne, Frederick A. Masoudi, Jeptha P. Curtis, Matthew M. Zipse, Amneet Sandhu, Jonathan C. Hsu, Pamela N. Peterson

**Affiliations:** 1 Division of Cardiology, University of Colorado Anschutz Medical Campus, Aurora; 2 Section of Cardiovascular Medicine, Department of Medicine, Yale University School of Medicine, New Haven, Connecticut; 3 Center for Outcomes Research and Evaluation, Yale-New Haven Hospital, New Haven, Connecticut; 4 Section of Cardiac Electrophysiology, Division of Cardiology, University of California, San Diego, La Jolla; 5 Division of Cardiology, Denver Health Hospital, Denver, Colorado

## Abstract

**Importance:**

Frequent right ventricular (RV) pacing can cause and exacerbate heart failure. Cardiac resynchronization therapy (CRT) has been shown to be associated with improved outcomes among patients with reduced left ventricular ejection fraction who need frequent RV pacing, but the patterns of use of CRT vs dual chamber (DC) devices and the associated outcomes among these patients in clinical practice is not known.

**Objective:**

To assess outcomes, variability in use of device type, and trends in use of device type over time among patients undergoing implantable cardioverter defibrillator (ICD) implantation who were likely to require frequent RV pacing but who did not have a class I indication for CRT.

**Design, Setting, and Participants:**

This retrospective cohort study used the National Cardiovascular Data Registry (NCDR) ICD Registry. A total of 3100 Medicare beneficiaries undergoing first-time implantation of CRT defibrillator (CRT-D) or DC-ICD from 2010 to 2016 who had a class I or II guideline ventricular bradycardia pacing indication but not a class I indication for CRT were included. Data were analyzed from August 2018 to October 2019.

**Exposures:**

Implantation of a CRT-D or DC-ICD.

**Main Outcomes and Measures:**

All-cause mortality, heart failure hospitalization, and complications were ascertained from Medicare claims data. Multivariable Cox proportional hazards models and Fine-Gray models were used to evaluate 1-year mortality and heart failure hospitalization, respectively. Multivariable logistic regression was used to evaluate 30-day and 90-day complications. All models accounted for clustering. The median odds ratio (MOR) was used to assess variability and represents the odds that a randomly selected patient receiving CRT-D at a hospital with high implant rates would receive CRT-D if they had been treated at a hospital with low CRT-D implant rates.

**Results:**

A total of 3100 individuals were included. The mean (SD) age was 76.3 (6.4) years, and 2500 (80.6%) were men. The 1698 patients (54.7%) receiving CRT-D were more likely than those receiving DC-ICD to have third-degree atrioventricular block (828 [48.8%] vs 432 [30.8%]; *P* < .001), nonischemic cardiomyopathy (508 [29.9%] vs 255 [18.2%]; *P* < .001), and prior heart failure hospitalizations (703 [41.4%] vs 421 [30.0%]; *P* < .001). Following adjustment, CRT-D was associated with lower 1-year mortality (hazard ratio [HR], 0.70; 95% CI, 0.57-0.87; *P* = .001) and heart failure hospitalization (subdistribution HR, 0.77; 95% CI, 0.61-0.97; *P* = .02) and no difference in complications compared with DC-ICD. Hospital variation in use of CRT was present (MOR, 2.00), and the use of CRT in this cohort was higher over time (654 of 1351 [48.4%] in 2010 vs 362 of 594 [60.9%] in 2016; *P* < .001).

**Conclusions and Relevance:**

In this cohort study of older patients in contemporary practice undergoing ICD implantation with a bradycardia pacing indication but without a class I indication for CRT, CRT-D was associated with better outcomes compared with DC devices. Variability in use of device type was observed, and the rate of CRT implantation increased over time.

## Key Points

**Question:**

Among patients undergoing implantable cardioverter defibrillator (ICD) implantation who are likely to require frequent right ventricular pacing, what are the outcomes of cardiac resynchronization therapy (CRT) compared with a dual chamber device, what is the variability in use of device type, and what are the trends in use of device type over time?

**Findings:**

In this cohort study of 3100 Medicare patients undergoing first-time ICD implantation with a ventricular bradycardia pacing indication, the use of CRT was associated with a lower risk of heart failure hospitalization and 1-year mortality without an increase in procedural complications compared with a dual chamber device. Variability in use of device type was observed, and the rate of CRT implantation increased over time.

**Meaning:**

In this study, CRT was associated with better outcomes than dual chamber devices among adult patients.

## Teaser

This cohort study assesses outcomes, variability in use of device type, and trends in use of device type over time among patients undergoing implantable cardioverter defibrillator implantation.

## Introduction

Frequent right ventricular (RV) pacing may be deleterious, given that it results in electrical dyssynchrony, can exacerbate symptoms of heart failure, and is associated with incident atrial fibrillation.^1,2^ Cardiac resynchronization therapy (CRT) improves morbidity and mortality among patients with low left ventricular ejection fraction (LVEF) and electrical dyssynchrony.^3^ However, among patients without a class I indication for CRT but with an indication for ventricular pacing, it is unclear who might derive benefit from CRT vs a traditional dual chamber (DC) device.

The Biventricular Pacing for Atrioventicular Block and Systolic Dysfunction (BLOCK-HF) randomized clinical trial evaluated the use of CRT in a broader group of patients with reduced left ventricular systolic function with frequent anticipated ventricular pacing and found a lower incidence of a combined end point of time to death from any cause, heart failure visit, or increase in the left ventricular end-systolic volume index among patients randomized to CRT compared with patients receiving a traditional DC system.^4^ While the results of this trial were published in 2013, it took until 2018 for an update and change in guidelines. Based on this trial, the 2018 American College of Cardiology, American Heart Association, and the Heart Rhythm Society guidelines include a class IIa recommendation for CRT among patients with an LVEF of 36% to 50% and an indication for permanent pacing who are expected to require frequent ventricular pacing.^5^ The extent of practice variation and, importantly, outcomes among real-world patients with anticipated frequent ventricular pacing during this time frame is unknown.

Patients undergoing implantable cardioverter defibrillator (ICD) implantation frequently have conduction disturbances and an indication for ventricular pacing. The National Cardiovascular Data Registry (NCDR) ICD Registry provides a unique opportunity to analyze this group of patients. Accordingly, we sought to examine the trends, variability, and outcomes in the use of CRT defibrillator (CRT-D) and DC-ICD devices among patients undergoing ICD implantation from 2010 to 2016 with a ventricular pacing indication who did not have a class I indication for CRT based on LVEF.

## Methods

### Data Source

Data were obtained from the NCDR ICD Registry, the details of which have been reported previously.^6^ The ICD Registry was designed to satisfy the requirements of the 2005 Centers for Medicare & Medicaid Services (CMS) coverage with evidence decision for primary prevention ICD implantation. In addition to expanded coverage, CMS mandated that data on all Medicare primary prevention implantations be entered into the NCDR ICD Registry until the data collection requirement ended on February 15, 2018.^6^

The registry collects data from more than 1500 hospitals in the United States and included more than 1.3 million records as of the end of 2014.^7,8,9^ Although CMS mandated that only primary prevention devices be entered into the registry, it is estimated that 90% of all ICD implantations are documented.^7^ The registry uses a standardized data set and definitions, has requirements in place to ensure uniform data entry and transmission, and is subject to data quality checks.^10^ All data submissions are evaluated for errors and completeness. This information is summarized in an automated report that is sent to the participants after each data submission. The NCDR audit program, which includes hospital medical record reviews and blinded data abstractions, serves as an additional mechanism to assess the accuracy of the data and enables participants to identify areas for improved data entry. Outcomes following discharge from the index hospitalization (including mortality, heart failure hospitalization, and complications) were obtained by linking NCDR registry files with Medicare inpatient fee-for-service claims, as previously described.^11^

Statistical analysis was approved and completed by the Yale Center for Outcomes Research and Evaluation. The initial analysis was performed on August 27, 2018, and completed in October 2019. Analyses of the NCDR ICD Registry are performed under an institutional review board approval by Yale University, with a waiver of informed consent because of the study design. This study followed the Strengthening the Reporting of Observational Studies in Epidemiology (STROBE) reporting guideline.

### Study Population

All patients undergoing initial implantation of a transvenous DC-ICD or CRT-D in the NCDR ICD Registry from April 2010 to March 2016 with a class I or II guideline ventricular bradycardia pacemaker indication (ie, second- or third-degree atrioventricular block or a PR interval ≥300 ms) were included. Outcomes were ascertained among patients who could be linked to Medicare through 2014. Patients were excluded if they had a class I indication for CRT (ie, LVEF ≤35%; left bundle branch block with a QRS interval ≥150 ms; sinus rhythm; and New York Heart Association (NYHA) II, III, or ambulatory IV symptoms). Patients with a previous pacemaker, ICD, coronary sinus or left ventricular lead, or epicardial lead were excluded. Using an established method, eligible patients were matched to Medicare claims data based on indirect identifiers, including age, sex, admission date or procedure date, and hospital CMS certification number.^11^

### Outcomes

The outcomes analysis was 3-fold: the long-term outcomes included time to death from any cause and heart failure hospitalization. Short-term outcomes of interest included device related complications (pneumothorax or hemothorax at 30 days, hematoma requiring transfusion or surgical evacuation at 30 days, pericardial tamponade requiring pericardiocentesis at 30 days, mechanical complications with system revisions at 90 days, device-related infection at 90 days, ICD replacement at 90 days, or death at 30 days). Outcomes were ascertained from Medicare claims and assessed either to the extent they were available or until December 2014.

### Statistical Analysis

Continuous variables are presented as means and SDs and categorical variables as numbers and proportions. Patient, hospital, and clinician factors were compared between patients undergoing DC-ICD and CRT-D using χ^2^ test for categorical variables and *t* test for continuous variables. The factors associated with CRT-D use were identified through multivariable logistic regression models, which included all covariates.

The associations between the type of device implanted and outcomes were assessed using multivariable Cox proportional hazards models for mortality and Fine-Gray models for heart failure hospitalization (subdistribution hazard ratio [HR]) to account for competing risk of death. Robust sandwich variance estimates were used in both models to account for clustering of patients within hospitals. The proportional hazard assumptions were tested by including the interaction terms of covariates and time. A hierarchical logistic regression model was used for complications (odds ratio [OR]). Outcomes were evaluated based on time to event for mortality and heart failure hospitalization, with device type as the key independent variable and adjusting for patient demographic characteristics, comorbidities, hospital characteristics, and geographic region. Covariates were chosen based on prior literature and clinical experience.^12,13,14,15,16^ Specifically, covariates in the models included patient demographic characteristics (age, sex, race); baseline clinical characteristics (hospitalization reason [admitted for procedure vs not], NYHA class, atrial fibrillation or flutter, ventricular tachycardia, nonischemic dilated cardiomyopathy, ischemic cardiomyopathy, previous valvular surgery, cerebrovascular disease, chronic lung disease, diabetes, hypertension, kidney failure and receiving dialysis, mean ejection fraction, mean QRS duration, paced cardiac rhythm, second- or third-degree atrioventricular block, left bundle branch block, mean creatinine level, and mean systolic blood pressure); physician training; hospital characteristics (geographic location, profit type, urban or rural location, and teaching status); and device type including primary or secondary prevention indication. The prevalence of missing values was very low for all variables (<1%), except for LVEF (46 [1.5%]), hospital profit type (93 [3%]), and implanter specialty training (527 [17%]). Missing values were imputed to avoid case-wise deletion. Missing continuous variables were imputed with the overall median value. For categorical variables, we imputed the missing values using the most common category of each variable. We defined the test threshold of error at *P* = .05, and all tests were 2-tailed. All analyses were conducted with SAS version 9.3 (SAS Institute).

To quantify the extent to which practice variation was explained by hospital-level effects, the hospital-specific median OR (MOR) was calculated using a validated method for the entire cohort and for each year.^17^ Hierarchical logistic regression models were used to determine the between-hospital variance of CRT-D use, accounting for clustering of patients within hospitals, and the MOR was calculated. The MOR represents the odds that a randomly selected patient receiving a CRT-D at a hospital with high implant rates would be implanted compared with receiving care at a hospital with low CRT-D implant rates. This analysis was performed among patients implanted from April 2010 to March 2016. We included patients who were not matched to Medicare claims data in the analysis for institutional variation and temporal trends. Temporal changes in device type implant were assessed using the Cochran-Armitage trend test.

## Results

### Study Population

Within the NCDR, 10 483 patients aged 65 years or older underwent a primary or secondary prevention ICD with a bradycardia pacing indication (second- or third-degree atrioventricular block or a PR interval of ≥300 ms) and could be linked to Medicare data. There were 7383 patients (70.4%) excluded from our final analysis owing to class I indication for CRT (2181 [29.5%]) and epicardial left ventricular lead or prior pacemaker (5202 [70.5%]), resulting in a study cohort of 3100 patients.

### Patient Demographic Characteristics

The patient population is described in Table 1. The mean (SD) age of the population was 76.3 (6.4) years, and 2500 (80.6%) were men. More than half of patients had ischemic heart disease (2167 [69.9%]). The mean (SD) LVEF was 31.2% (11.8%). More patients had third-degree atrioventricular block (1260 [40.6%]) than second-degree atrioventricular block (965 [31.1%]).

**Table 1.** Baseline Characteristics Among Patients Undergoing DC-ICD and CRT-D

Characteristic	No. (%)			*P* value
	All (N = 3100)	DC (n = 1402)	CRT (n = 1698)	
Age				
Mean (SD)	76.3 (6.4)	76.4 (6.5)	76.2 (6.3)	.38
65-74	1281 (41.3)	566 (40.4)	715 (42.1)	.60
75-84	1473 (47.5)	675 (48.1)	798 (47.0)	
>84	346 (11.2)	161 (11.5)	185 (10.9)	
Women	600 (19.4)	304 (21.7)	296 (17.4)	.003
Latino ethnicity	121 (3.9)	53 (3.8)	68 (4.0)	.75
Race				
White	2744 (88.5)	1223 (87.2)	1521 (89.6)	.10
Black	285 (9.2)	141 (10.1)	144 (8.5)	
Other^a^	71 (2.3)	38 (2.7)	33 (1.9)	
Second-degree atrioventricular block	965 (31.1)	465 (33.2)	500 (29.4)	.03
Third-degree atrioventricular block	1260 (40.6)	432 (30.8)	828 (48.8)	<.001
PR interval				
Mean (SD), ms	289.0 (90.1)	288.7 (87.4)	289.2 (93.0)	.91
≥300 ms	1098 (35.4)	601 (42.9)	497 (29.3)	<.001
Nonischemic cardiomyopathy	763 (24.6)	255 (18.2)	508 (29.9)	<.001
ICD indication				
Primary prevention	2335 (75.3)	879 (62.7)	1456 (85.7)	<.001
Secondary prevention	765 (24.7)	523 (37.3)	242 (14.3)	
LVEF %				
Mean (SD)	31.2 (11.8)	35.7 (13.9)	27.6 (8.0)	<.001
<25	698 (22.5)	212 (15.1)	486 (28.6)	<.001
25-35	1746 (56.3)	679 (48.4)	1067 (62.8)	
36-55	459 (14.8)	331 (23.6)	128 (7.5)	
>55%	151 (4.9)	145 (10.3)	6 (0.4)	
History of HF				
No	623 (20.1)	477 (34.0)	146 (8.6)	<.001
Yes, no prior HF hospitalization	1345 (43.4)	502 (35.8)	843 (49.6)	
Yes, prior HF hospitalization	1124 (36.3)	421 (30.0)	703 (41.4)	
Ventricular tachycardia	1117 (36.0)	674 (48.1)	443 (26.1)	<.001
Cardiac arrest	421 (13.6)	267 (19.0)	154 (9.1)	<.001
Syncope	756 (24.4)	405 (28.9)	351 (20.7)	<.001
Ischemic heart disease	2167 (69.9)	974 (69.5)	1193 (70.3)	.58
Prior MI	1699 (54.8)	781 (55.7)	918 (54.1)	.65
Prior PCI	1005 (32.4)	472 (33.7)	533 (31.4)	.40
Prior CABG	1256 (40.5)	555 (39.6)	701 (41.3)	.32
Primary valvular disease	497 (16.0)	203 (14.5)	294 (17.3)	.07
NYHA class				
I	437 (14.1)	345 (24.6)	92 (5.4)	<.001
II	878 (28.3)	562 (40.1)	316 (18.6)	
III	1631 (52.6)	449 (32.0)	1182 (69.6)	
IV	144 (4.6)	39 (2.8)	105 (6.2)	
Atrial fibrillation or flutter	1162 (37.5)	483 (34.5)	679 (40.0)	.007
Cerebrovascular disease	613 (19.8)	284 (20.3)	329 (19.4)	.62
Diabetes	1457 (47.0)	643 (45.9)	814 (47.9)	.22
Kidney failure, receiving dialysis	134 (4.3)	67 (4.8)	67 (3.9)	.28
Chronic lung disease	690 (22.3)	311 (22.2)	379 (22.3)	.44
Sleep apnea	400 (12.9)	176 (12.6)	224 (13.2)	.60
Hypertension	2667 (86.0)	1218 (86.9)	1449 (85.3)	.23
QRS duration				
<120 ms	1044 (33.7)	696 (49.6)	348 (20.5)	<.001
≥120 ms	2056 (66.3)	706 (50.4)	1350 (79.5)	
Abnormal intraventricular conduction	2067 (66.7)	733 (52.3)	1334 (78.6)	<.001
RBBB	944 (30.5)	378 (27.0)	566 (33.3)	<.001
LBBB	602 (19.4)	151 (10.8)	451 (26.6)	<.001
Hemoglobin, mean (SD), g/dL	12.4 (2.0)	12.3 (2.0)	12.5 (2.0)	.04
Creatinine, mean (SD), mg/dL	1.5 (1.3)	1.5 (1.4)	1.5 (1.2)	.68
BUN, mean (SD), mg/dL	27.7 (14.6)	26.6 (14.0)	28.6 (15.0)	<.001
Sodium, mean (SD), mEq/L	138.3 (4.3)	138.3 (4.8)	138.3 (3.7)	.94
Potassium, mean (SD), mEq/L	4.2 (0.5)	4.2 (0.5)	4.2 (0.5)	.22
BNP, mean (SD), pg/mL	1242.7 (1510.2)	1251.4 (1572.2)	1236.4 (1465.9)	.89

Abbreviations: BNP, brain-type natriuretic peptide; BUN, blood urea nitrogen; CABG, coronary artery bypass grafting; CRT, cardiac resynchronization therapy; DC, dual chamber; HF, heart failure; ICD; implantable cardioverter defibrillator; LBBB, left bundle branch block; LVEF, left ventricular ejection fraction; MI, myocardial infarction; NYHA, New York Heart Association; PCI, percutaneous coronary intervention; RBBB, right bundle branch block.

SI conversion factors: To convert BNP to nanograms per liter, multiply by 1.0; BUN to millimoles per liter, multiply by 0.357; creatinine to micromoles per liter, multiply by 88.4; hemoglobin to grams per liter, multiply by 10.0; potassium and sodium to millimoles per liter, multiply by 1.0.

^a^ Other includes Asian, American Indian or Alaskan Native, and Native Hawaiian or Pacific Islander.

More patients underwent CRT-D (1698 [54.8%]) than DC-ICD (1402 [45.2%]) (*P* < .001). Patients undergoing DC-ICD, compared with those undergoing CRT-D, were more likely to be women (304 [21.7%] vs 296 [17.4%]; *P* = .003), have a higher mean (SD) LVEF (35.7% [13.9%] vs 27.6% [8.0%]; *P* < .001), have had ventricular tachycardia (674 [48.1%] vs 443 [26.1%]; *P* < .001), and have a shorter QRS duration (<120 ms: 696 [49.6%] vs 348 [20.5%]; *P* < .001). Patients undergoing CRT-D were more likely that those undergoing DC-ICD to have third-degree atrioventricular block (828 [48.8%] vs 432 [30.8%]; *P* < .001), nonischemic cardiomyopathy (508 [29.9%] vs 255 [18.2%]; *P* < .001), a primary prevention indication (1456 [85.7%] vs 879 [62.7%]; *P* < .001), prior heart failure hospitalization (703 [41.4%] vs 421 [30.0%]; *P* < .001), more advanced NYHA class (class III: 1182 [69.6%] vs 449 [32.0%]; *P* < .001), and a right bundle branch block (566 [33.3%] vs 378 [27.0%]; *P* < .001) or left bundle branch block (451 [26.6%] vs 151 [10.8%]; *P* < .001).

The factors associated with CRT-D use in a multivariable model are shown in Table 2. The highest OR for use of CRT was among patients with NYHA class III symptoms (4.14; 95% CI, 2.86-5.98), followed by NYHA class IV (4.03; 95% CI, 2.27-7.13), QRS duration of 120 ms or greater (2.72; 95% CI, 2.11-3.50), and third-degree atrioventricular block (1.84; 95% CI, 1.46-2.31).

**Table 2.** Factors Associated With Cardiac Resynchronization Therapy Defibrillator Placement

Factor	OR (95% CI)	*P* value
Age, y		
65-74	1 [Reference]	NA
75-84	1.00 (0.82-1.23)	.69
>84	0.88 (0.63-1.22)	
Women	0.71 (0.54-0.91)	.01
Latino ethnicity	0.83 (0.51-1.35)	.45
Race		
White	1 [Reference]	NA
Black	0.70 (0.50-0.99)	.05
Other	0.62 (0.32-1.20)	
Second-degree atrioventricular block	1.17 (0.93-1.47)	.17
Third-degree atrioventricular block	1.84 (1.46-2.31)	<.001
Nonischemic cardiomyopathy	1.37 (1.01-1.85)	.04
ICD indication		
Primary prevention	1 [Reference]	
Secondary prevention	0.67 (0.50-0.91)	.01
LVEF %	0.96 (0.95-0.97)	<.001
History of HF		
No	1 [Reference]	NA
Yes, no prior HF hospitalization	1.90 (1.39-2.60)	<.001
Yes, prior HF hospitalization	1.82 (1.30-2.53)	
Ventricular tachycardia	0.71 (0.57-0.89)	<.001
Cardiac arrest	0.72 (0.51-1.00)	.05
Syncope	0.85 (0.67-1.07)	.17
Ischemic heart disease	1.06 (0.77-1.46)	.72
Prior MI	0.84 (0.65-1.07)	.15
Prior PCI	1.11 (0.89-1.38)	.37
Prior CABG	1.16 (0.92-1.45)	.21
Primary valvular disease	0.94 (0.72-1.22)	.62
NYHA class		
I	1 [Reference]	NA
II	1.04 (0.72-1.50)	<.001
III	4.14 (2.86-5.98)	
IV	4.03 (2.27-7.13)	
Atrial fibrillation or flutter	1.13 (0.93-1.39)	.22
Cerebrovascular disease	0.86 (0.68-1.09)	.22
Diabetes	1.05 (0.86-1.29)	.60
Kidney failure, receiving dialysis	1.24 (0.71-2.18)	.45
Chronic lung disease	0.80 (0.64-1.01)	.06
Sleep apnea	1.06 (0.79-1.43)	.68
Hypertension	1.03 (0.78-1.35)	.86
QRS duration, ms		
<120	1 [Reference]	NA
≥120	2.72 (2.11-3.50)	<.001
Abnormal intraventricular conduction	1.89 (1.42-2.51)	<.001
RBBB	0.90 (0.69-1.17)	.42
LBBB	1.92 (1.40-2.63)	<.001
Operator ICD training		
EP cardiologist	1 [Reference]	NA
Thoracic surgeon	0.38 (0.18-0.80)	<.001
Non-EP cardiologist	0.39 (0.27-0.54)	
Other specialist	0.22 (0.15-0.33)	
Hospital type		
Government	1 [Reference]	NA
Private	0.89 (0.32-2.50)	.96
University	0.93 (0.32-2.74)	
Location		
Rural	1 [Reference]	NA
Suburban	1.34 (0.95-1.89)	.24
Urban	1.22 (0.88-1.70)	
Region		
Midwest	1 [Reference]	NA
Northeast	0.88 (0.62-1.26)	.11
South	0.96 (0.73-1.25)	
West	1.39 (0.98-1.98)	
Teaching hospital	1.18 (0.92-1.50)	.19

Abbreviations: CABG, coronary artery bypass grafting; EP, electrophysiology; HF, heart failure; ICD, implantable cardioverter defibrillator; LBBB, left bundle branch block; LVEF, left ventricular ejection fraction; MI, myocardial infarction; NA, not applicable; NYHA, New York Heart Association; OR, odds ratio; PCI, percutaneous coronary intervention; RBBB, right bundle branch block.

### Outcomes

**Figure 1.**
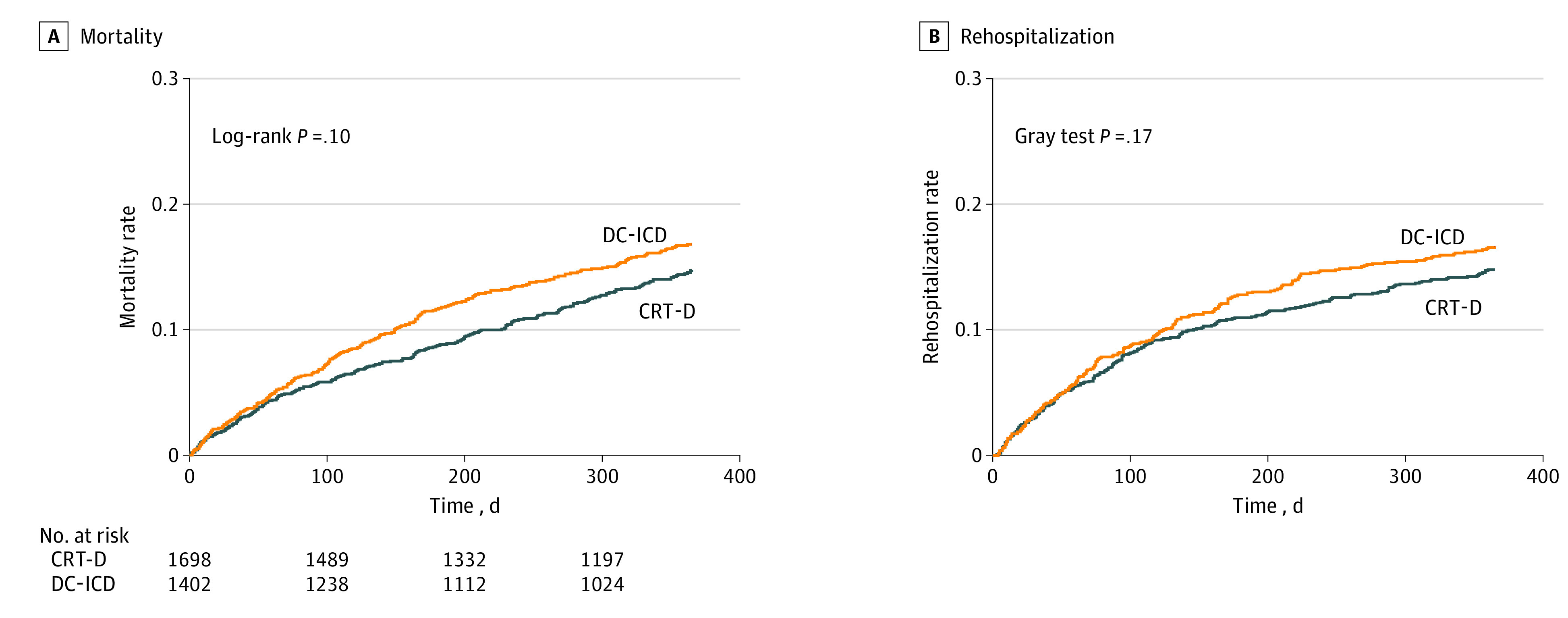
Unadjusted Outcomes Stratified by Cardiac Resynchronization Therapy Defibrillator (CRT-D) or Dual Chamber Implantable Cardioverter Defibrillator (DC-ICD)

There was no evidence of difference between CRT-D and DC-ICD in the unadjusted incidence of device-related complications (184 [10.8%] vs 131 [9.3%]; *P* = .17), 1 year incidence of death (13.3% vs 15.7%; *P* = .05), or 1 year incidence of heart failure hospitalization (13.5% vs 15.7%; *P* = .09) (Figure 1 and eTable in the Supplement). Following adjustment, the incidence of complications between the groups was not different (OR, 1.26; 95% CI, 0.93-1.70; *P* = .12); however, the risks of death (HR, 0.70; 95% CI, 0.57-0.87; *P* = .001) and heart failure hospitalization (subdistribution HR, 0.77; 95% CI, 0.61-0.97; *P* = .02) were lower among patients undergoing implantation of a CRT-D compared with those undergoing an implantation of a DC-ICD.

### Institutional Variation and Temporal Trends

**Figure 2.**
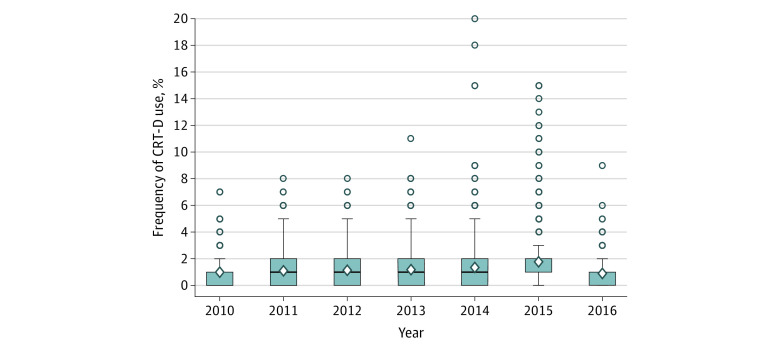
Hospital Frequency Use of Cardiac Resynchronization Therapy Defibrillator (CRT-D) The diamond represents the mean, the box represents the 25th and 75th percentile, and the median is the line between 25th and 75th. Whisker bars indicate 95% CI, and circles, outliers.

**Figure 3.**
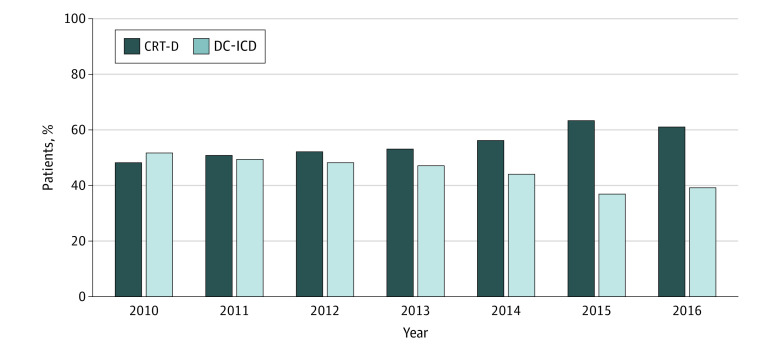
Temporal Trends of Cardiac Resynchronization Therapy Defibrillator (CRT-D) or Dual Chamber Implantable Cardioverter Defibrillator (DC-ICD)

Hospital level variation in the use of DC-ICD or CRT-D was present among this patient cohort. The MOR for the entire cohort was 2.00, which varied across the study years: 2.14 in 2010, 1.98 in 2011, 2.02 in 2012, 2.02 in 2012, 1.83 in 2013, 1.84 in 2014, 1.93 in 2015. Figure 2 illustrates hospital frequency use of CRT-D. Across the study years, a trend toward use of more CRT-D was observed (654 of 1351 [48.4%] in 2010 to 362 of 594 [60.9%] in 2016; *P* < .001) (Figure 3).

## Discussion

This study evaluated the use and outcomes of DC-ICD and CRT-D implantations among patients undergoing first-time ICD implantation with a bradycardia pacing indication and without a class I indication for CRT among a national cohort of patients treated in clinical practice. After adjustment, CRT use was associated with a lower risk of mortality and hospitalization for heart failure without a higher risk of complications. Institutional variation in the type of device implanted was present, which was not associated with patient characteristics. Finally, there was a temporal trend toward higher use of CRT during the 5-year study period. These results have important implications for the standard of care for patients receiving an ICD who have a pacing indication.

To our knowledge, this is the first real-world study to evaluate the association of device therapy and outcomes among patients with a ventricular pacing indication but without a class I indication for CRT. Our findings are consistent with prior clinical trials evaluating the effect of frequent RV pacing on clinical outcomes. Among the first randomized trials showing forced RV pacing was detrimental was the Dual Chamber and VVI Implantable Defibrillator (DAVID) trial, which randomized patients with reduced LVEF without a bradycardia pacing indication to DC rate responsive pacing at 70 beats per minute (bpm) or back up ventricular pacing at 40 bpm. Patients with frequent RV pacing had a detectably higher incidence of the composite end point of death or heart failure hospitalization.^18^ However, this trial did not examine patients requiring frequent ventricular pacing. The BLOCK-HF trial randomized patients with a bradycardia pacing indication and mildly reduced LVEF to CRT or DC pacing and demonstrated a detectable improvement in the primary end point for patients with CRT.^4^

Consistent with prior clinical trials, in an analysis of contemporary real-world patients, we observed a lower incidence of adverse outcomes among the patients undergoing CRT-D compared with those with RV pacing (DC-ICD), which continued beyond a year of follow up. While the outcome of death was not detectable in BLOCK-HF, we observed an association between CRT-D and death despite the fact that patients in the current study tended to be older, with lower LVEF and greater burden of comorbidities, including prior myocardial infarction, hypertension, and diabetes. However, the findings of the present study are observational and thus the observed associations cannot be interpreted as causal.

We observed variation in the use of CRT or DC-ICD implantations among this cohort. For example, patients undergoing CRT-D implantation were more likely to have advanced atrioventricular block and NYHA class III or IV, prior heart failure hospitalization, and wider QRS duration. This is in concordance with prior literature showing improvement in heart failure symptoms with CRT and the need for chronic pacing.^3,4^ In contrast, patients with a very long PR interval (≥300ms) were more likely to undergo DC-ICD implantation. While efforts to minimize ventricular pacing in patients with a long PR interval and DC devices is possible with ventricular pacing avoidance algorithms, this has limitations regarding upper-rate behavior and without causing pacemaker syndrome. As such, these patients were included in the BLOCK-HF trial as likely needing chronic RV pacing and should be considered for CRT at the time of implantation.^4^

We also observed hospital-level variation in the type of device implanted, which was independent of patient characteristics. Plausible explanations for this include familiarity with the more technically challenging LV lead placement or regional culture regarding the value of CRT. Interestingly, nonelectrophysiology implanting clinicians were more likely to implant a DC-ICD, which can be seen to provide a basis for lack of specific training or familiarity to implanting an LV lead. Describing this type of variation in care is an important first step in understanding the extent to which patient factors, compared with institutional factors, are associated with variation. Clustering of device therapy (ie, DC-ICD use more often at some hospitals) could provide opportunities for quality improvement programs to address hospital-level differences associated with variation.

A higher proportion of CRT-D implantations was noted among this cohort across the study years. The BLOCK-HF trial was published in 2013, and following this publication, there appears to have been an increase in the selection of CRT devices.^4^ This study provides a perspective on the pace of adoption of CRT among this patient population based on the publication of an important trial.

The updated 2018 guidelines on the evaluation and treatment of patients with bradycardia and cardiac conduction delay were the first to provide recommendations on the use of CRT among patients with mildly reduced LVEF. The document provides a class IIa indication for physiologic ventricular activation, including CRT or His bundle pacing, among patients with LVEF between 36% and 50% who are expected to require ventricular pacing.^5^ This is supported by level of evidence B-R (moderate-quality evidence from randomized trials) which is based on data from BLOCK-HF. While the current study has limitations inherent in observational research, including inability to determine causality, it adds to the limited data that support the more frequent use of CRT among patients with mildly reduced LVEF and anticipated high RV pacing requirement.

Further randomized and real-world investigations are warranted to both confirm the findings seen in BLOCK-HF, which would strengthen the recommendation to a class I indication among those with reduced LVEF, and to potentially expand the use of CRT among patients with normal LVEF and the need for frequent RV pacing, particularly those with evidence of electrical dyssynchrony or wide QRS intervals at baseline. Additionally, His or left bundle pacing may develop into an alternative or replacement to the traditional CRT system, and well-designed randomized trials are needed. Hospital variation in use of different modalities of cardiac resynchronization may be associated with procedural volumes and outcomes, and additional analysis evaluating these associations is warranted. Ultimately, the risk of implanting a device with higher procedural complexity and reduction in battery longevity requiring more frequent generator changes needs to be weighed against the potential benefits.

### Limitations

This study has limitations that should be considered in the interpretation of this study. First, because this study is observational, we cannot exclude the possibility that unmeasured confounding variables influenced the association between device type and outcomes. Second, the amount of RV pacing in follow-up is not available in the ICD Registry; an understanding of this parameter would provide additional mechanistic insights into the association between CRT-D and outcomes. However, this is commonly the case in clinical practice: clinicians need to use their best judgement on what the expected RV pacing burden will be with the limited information available at the time of device implantation. Additionally, specific information about device interventions, including ICD shocks or antitachycardia pacing, is not available in the ICD Registry. Third, the position of the RV lead has been implicated in the development of cardiomyopathy, and this location is not available in the registry. Fourth, while the NCDR is a voluntary reporting system that encompasses most ICD implantations, it is not all inclusive, and it is possible that the difference in device implantation varies significantly among centers that do not report to the NCDR.

## Conclusions

In this cohort study of older patients in contemporary practice who underwent ICD implantation with a bradycardia pacing indication but without a class I indication for CRT, CRT use was associated with better outcomes, including all-cause mortality and heart failure hospitalization. These findings are consistent with the results of the BLOCK-HF trial.

## Notes

Supplement. **eTable.** Association of Covariates With 1-Year Mortality
