# Supplementary material for: Use and Outcomes of Dual Chamber or Cardiac Resynchronization Therapy Defibrillators Among Older Patients Requiring Ventricular Pacing in the National Cardiovascular Data Registry Implantable Cardioverter Defibrillator Registry
Source: JAMA Netw Open. 2021 Jan 26;4(1):e2035470. doi: 10.1001/jamanetworkopen.2020.35470 (PMC7838925; doi:10.1001/jamanetworkopen.2020.35470)
Supplement: Supplement. — eTable. Association of Covariates With 1-Year Mortality [file jamanetwopen-e2035470-s001.pdf]

Supplemental Online Content

Borne RT, Masoudi FA, Curtis JP, et al. Use and outcomes of dual chamber or cardiac resynchronization therapy defibrillators among older patients requiring ventricular pacing in the National Cardiovascular Data Registry Implantable Cardioverter Defibrillator Registry. *JAMA Netw Open*. 2021;4(1):e2035470. doi:10.1001/jamanetworkopen.2020.35470

eTable. Association of Covariates With 1-Year Mortality

This supplemental material has been provided by the authors to give readers additional information about their work.

© 2021 Borne RT et al. *JAMA Network Open.*

eTable. Association of Covariates With 1-Year Mortality

**1 year mortality**		
**Effects**	**Hazard Ratio (95%CI)**	**P value**
CRT-D	0.704 (0.571 - 0.868)	0.001
Age	1.057 (1.041 - 1.074)	<.001
Gender (Female)	1.448 (1.144 - 1.832)	0.002
Ethnicity (Latino)	1.113 (0.664 - 1.866)	0.68
Race		
White	ref	
Black	1.166 (0.858 - 1.585)	0.58
Other	1.104 (0.683 - 1.783)	.
Admitted for this procedure	0.610 (0.484 - 0.770)	.
NYHA class		
I	ref	
II	1.016 (0.711 - 1.452)	<.001
III	1.421 (1.003 - 2.013)	.
IV	2.398 (1.491 - 3.857)	.
Atrial Fibrillation/Atrial Flutter	1.205 (0.992 - 1.462)	0.06
Ventricular Tachycardia	1.228 (0.989 - 1.526)	0.06
Non-Ischemic Cardiomypathy	0.709 (0.553 - 0.910)	0.01
Primary valvular disease	1.217 (0.950 - 1.558)	0.12
Cerebrovascular Disease	1.356 (1.089 - 1.690)	0.01
Chronic Lung Disease	1.340 (1.091 - 1.646)	0.01
Diabetes	1.351 (1.104 - 1.654)	0.003
Hypertension	0.920 (0.691 - 1.225)	0.57
Renal Failure-Dialysis	2.295 (1.579 - 3.334)	<.001
EF %	0.983 (0.974 - 0.992)	0.002
QRS duration		
<120	ref	
≥120	1.206 (0.976 - 1.492)	0.08
Second or third degree heart block	1.140 (0.908 - 1.431)	0.26
LBBB	0.615 (0.458 - 0.825)	0.001
Creatinine mg/dL	1.081 (1.035 - 1.130)	<0.001
Systolic BP	0.993 (0.989 - 0.997)	0.002
Operator ICD training		
EP cardiologists	ref	
Thoracic surgeons	0.562 (0.235 - 1.345)	0.45
Non-EP cardiologists	0.837 (0.591 - 1.185)	.
Other specialists	0.961 (0.700 - 1.320)	.

© 2021 Borne RT et al. *JAMA Network Open.*

Hospital type		
Government	ref	
Private	0.588 (0.246 - 1.405)	0.47
University	0.569 (0.229 - 1.416)	.
Location		
Rural	ref	
Suburban	1.013 (0.737 - 1.392)	0.99
Urban	1.012 (0.751 - 1.365)	.
Region		
Midwest	ref	
Northeast	1.178 (0.861 - 1.610)	0.68
South	1.156 (0.889 - 1.504)	.
West	1.085 (0.774 - 1.521)	.
Teaching hospital	0.954 (0.769 - 1.184)	0.67

© 2021 Borne RT et al. *JAMA Network Open.*
